# Synthetic lethal analysis of *Caenorhabditis elegans *posterior embryonic patterning genes identifies conserved genetic interactions

**DOI:** 10.1186/gb-2005-6-5-r45

**Published:** 2005-04-11

**Authors:** L Ryan Baugh, Joanne C Wen, Andrew A Hill, Donna K Slonim, Eugene L Brown, Craig P Hunter

**Affiliations:** 1 Department of Molecular and Cellular Biology, Harvard University, Cambridge, MA 02138, USA; 2 Department of Genomics, Wyeth Research, Cambridge, MA 02140, USA; 3 Current address: Biology Division, California Institute of Technology, Pasadena, CA 91125; 4 Current address: Department of Computer Science, Tufts University, Medford, MA 02155

## Abstract

Phenotypic robustness is evidenced when single-gene mutations do not result in an obvious phenotype. It has been suggested that such phenotypic stability results from 'buffering' activities of homologous genes as well as non-homologous genes acting in parallel pathways. One approach to characterizing mechanisms of phenotypic robustness is to identify genetic interactions, specifically, double mutants where buffering is compromised. To identify interactions among genes implicated in posterior patterning of the *Caenorhabditis elegans *embryo, we measured synthetic lethality following RNA interference of 22 genes in 15 mutant strains. A pair of homologous T-box transcription factors (*tbx-8 *and *tbx-9*) is found to interact in both *C. elegans *and *C. briggsae*, indicating that their compensatory function is conserved. Furthermore, a muscle module is defined by transitive interactions between the MyoD homolog *hlh-1*, another basic helix-loop-helix transcription factor, *hnd-1*, and the MADS-box transcription factor *unc-120*. Genetic interactions within a homologous set of genes involved in vertebrate myogenesis indicate broad conservation of the muscle module and suggest that other genetic modules identified in *C. elegans *will be conserved.

## Abstract 2

To identify interactions among genes implicated in posterior patterning of the *C. elegans *embryo, synthetic lethality following RNA interference of 22 genes was measured in 15 mutant strains. A pair of homologous T-Box transcription factors was found to interact in both *C. elegans *and *C. briggsae*, indicating that their compensatory function is conserved.

## Background

Forward and reverse genetic screens in flies and worms indicate that most genes are not essential to the development or viability of the organism [1-4]. Two primary explanations for such phenotypic robustness to mutation have been offered: homologous gene products may directly compensate for one another's function, or indirect compensation of function may occur through regulatory networks via non-homologous genes acting in alternative pathways or feedback mechanisms [5,6]. In both *Saccharomyces cerevisiae *and *C. elegans*, genes with at least one homolog are less likely than unique genes to have a loss-of-function phenotype [7,8]. However, homology accounts for no more than two thirds of the observed phenotypic robustness to mutation in *S. cerevisiae *and even less in *C. elegans*, indicating a significant role of the regulatory network in genetic buffering [7,8]. By identifying gene disruptions that are viable in wild type but lethal in a specific mutant background, synthetic lethal screens can shed light on how regulatory networks buffer gene function [9]. Although genetic interactions are being mapped on a genome-wide scale in yeast [9], no such efforts have been reported for an animal system. We report here the use of existing mutants and RNA interference (RNAi) to assemble a synthetic lethal matrix in *C. elegans*. Our aim was to build on prior knowledge by focusing on a characterized set of genes likely to have interactions among them.

The C blastomere is one of five somatic founder blastomeres in the *C. elegans *embryo. Each founder blastomere produces a characteristic set of cell types via an invariant lineage; the C blastomere predominantly gives rise to muscle and epidermal cells [10]. Maternal and zygotic activities of the homeodomain protein PAL-1 specify the identity and maintain the development of the C-blastomere lineage [11,12]. We have identified genes whose expression depends either directly or indirectly on PAL-1 function ('PAL-1 targets') and shown that they affect specification, differentiation, and morphogenesis of C-lineage cells. PAL-1 targets include many transcription factors and based on their temporal and spatial expression patterns in wild type, we have proposed a model for their regulatory relationships [13]. However, RNAi of most PAL-1 targets does not result in a detectable phenotype [13]. Because the regulatory network specified by PAL-1 appears to function autonomously while controlling multiple, discrete developmental processes (that is, specification and morphogenesis of muscle and epidermal cells) [11,13,14], we reasoned that the network is organized in a modular fashion and that PAL-1 targets may have overlapping or compensatory functions. We set out to measure synthetic lethality for as many pairs of PAL-1 targets as possible, and we included three additional genes encoding transcriptional regulators (*lin-26*, *unc-62*, and *ceh-40*) whose expression patterns and phenotypes suggest that they may have genetic interactions with PAL-1 or its targets [15,16].

## Results and discussion

### A synthetic lethal matrix

**Figure 1:**
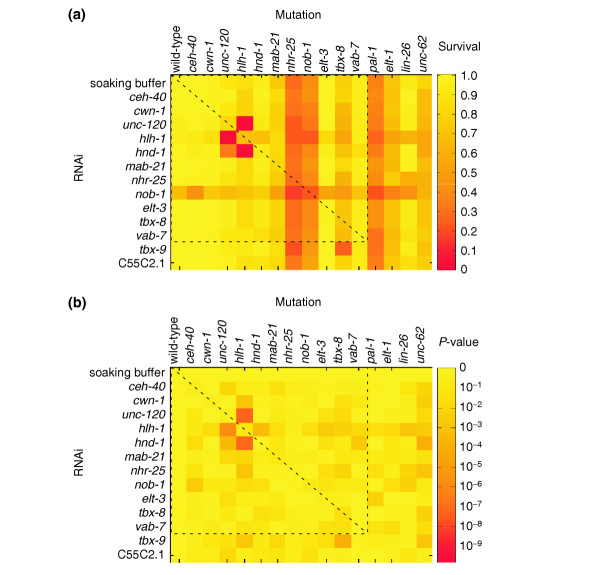
A synthetic lethal matrix of genes implicated in posterior patterning. **(a) **A heat map of survival (1 - % embryonic lethality) for RNAi of 13 genes (and a soaking buffer control) in 16 different genetic backgrounds. Values plotted are the average of two measurements (see Additional data file 1 for raw data). **(b) **A heat map of *P*-values corresponding to each test of synthetic lethality. A *t*-test was used with the null hypothesis that the survival of a given combination of RNAi and mutation is equal to the product of survivals of the RNAi in wild type and the mutation without RNAi (*P*-values can be found in Additional data file 1). A dashed gray line outlines the square (symmetric) part of the matrix and its diagonal in (a) and (b). RNAi of genes resulting in 100% embryonic lethality in wild type are not included in the matrix (*pal-1*, *elt-1*, *lin-26*, and *unc-62*). Mutant genotypes are listed in Table 1.

We began our analysis with 26 genes, 23 PAL-1 targets and three genes likely to have genetic interactions with PAL-1 targets. Mutations in 15 of these genes have been isolated, and appropriate strains were obtained from the *Caenorhabditis *Genetics Center (Table 1). To screen for synthetic lethal interactions, RNAi of the 13 nonessential genes was performed and embryonic lethality scored in each of the mutant strains as well as wild type to generate a synthetic lethal matrix (Figure 1 and Additional data file 1). However, RNAi of a given gene could not be used if it results in 100% lethality in wild type (*pal-1*, *elt-1*, *unc-62*, and *lin-26*), and so the matrix is not entirely symmetric. To reduce the penetrance of lethality following RNAi for these four genes we used a lower concentration of double-stranded RNA (dsRNA), but the results were variable (data not shown), and we decided that it was an unreliable approach. We also tried to assemble the matrix using exclusively RNAi, but we found that soaking worms in two different dsRNA molecules targeting two different genes at once to be unacceptably inefficient (Additional data file 2).

Given the effects of mutation alone without RNAi, and the effects of RNAi in wild type, it may not be obvious from the raw data which disruptions result in significantly more lethality when paired than when alone. Measurements of lethality were thus duplicated and a *t*-test was used to assign statistical significance to each of the interactions tested (Figure 1 and Additional data file 1). Seven genetic interactions were identified (*P *< 0.001) among six genes out of 195 total interactions tested. In addition to the genes shown in Figure 1, RNAi of the remaining genes for which no mutant was available was performed in all 16 strains. Because synthetic lethality was measured only a single time for each of these possible interactions, the data could not be subjected to statistical analysis. However, RNAi of these additional genes did not appear to elevate lethality in any of the genetic backgrounds tested (data not shown).

### Conservation of a genetic interaction

**Figure 2:**
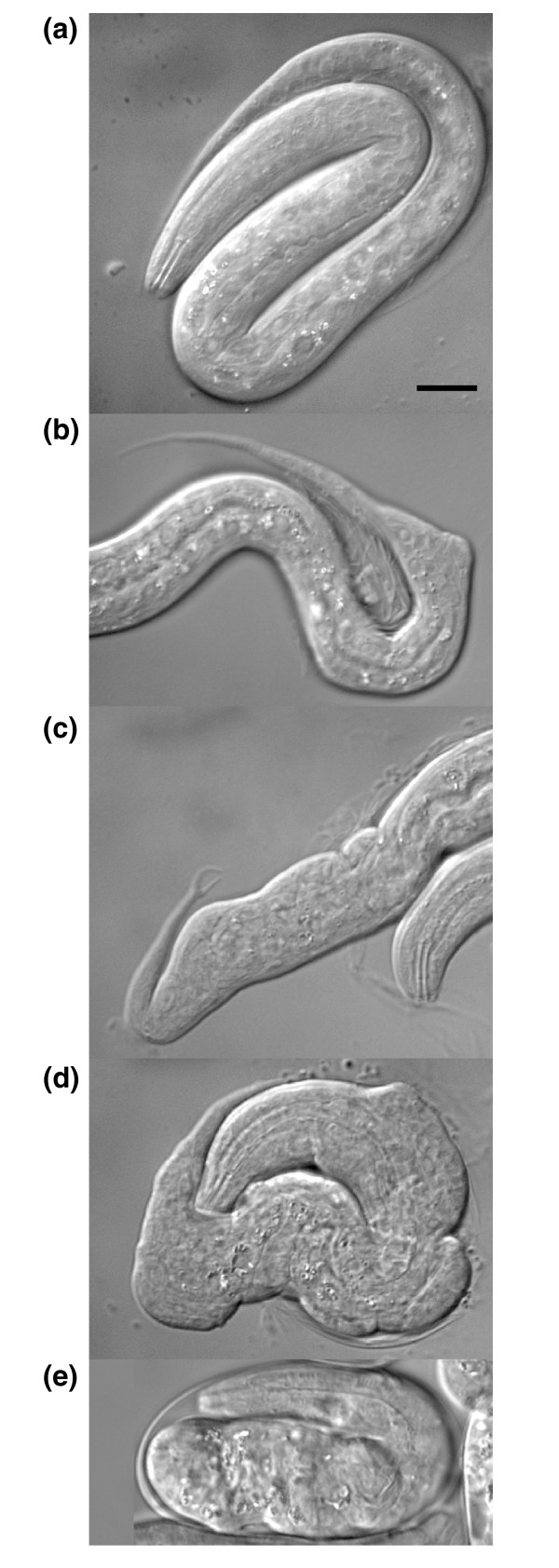
Synthetic lethal phenotypes of the homologous transcription factors *tbx-8 *and *tbx-9*. Recently hatched L1 larvae or an arrested embryo are shown for **(a) **wild-type, **(b) ***tbx-9(RNAi)*, **(c) ***tbx-8(ok656)*, and **(d, e) ***tbx-8(ok656); tbx-9(RNAi)*. Panels (b) to (e) reflect the increasing severity of the phenotype, with (b) and (c) providing examples of the posterior morphological defects observed at low frequency after disruption of *tbx-8 *or *tbx-9*. (d, e) Examples of the severe morphological defects observed at high frequency after disruption of both *tbx-8 *and *tbx-9*; (d) shows an individual that has hatched but will arrest as a larva, and (e) shows an embryo that has arrested before hatching (embryonic lethal). The anterior is cropped in (b) and (c). The scale bar in (a) equals 10 μm and applies to (a-e).

The T-box family of proteins regulates diverse aspects of animal development, including cell fate specification, differentiation, and morphogenesis [17]. The *C. elegans *genome contains 21 of these animal-specific genes, compared to 32 in *C. briggsae *and eight in *D. melanogaster *[18,19], suggesting duplication or retention within nematodes. *tbx-37 *and *tbx-38 *have been shown to redundantly control embryonic patterning of the *C. elegans *AB lineage [20], and our systematic approach identified *tbx-8 *and *tbx-9 *as a synthetic lethal pair. *tbx-8 *and *tbx-9 *are expressed in nearly identical temporal and spatial patterns in the early embryo, with the strongest expression detected in dorsal epidermal cells and posterior expression depending on *pal-1 *function [13,21,22]. *tbx-8(ok656)*, *tbx-8(RNAi)*, and *tbx-9(RNAi) *each result in a low frequency of embryonic lethality (less than 5%) with 1-10% of hatching larvae displaying posterior morphological defects (Figure 2) [21,22]. However, 50% of *tbx-8(ok656)*; *tbx-9(RNAi) *embryos fail to hatch and those that do display severe morphological defects and die as larvae (Figure 2). Phenotypic analysis demonstrates that *tbx-8 *and *tbx-9 *are required for intercalation of dorsal epidermal cells thus explaining the morphological defects observed following their disruption [22].

The overlapping function of *tbx-8 *and *tbx-9 *is not the residual result of a recent gene duplication event. The *C. briggsae *genome contains a pair of syntenic orthologs (by best reciprocal blastp match) [18], and as in *C. elegans*, the *C. briggsae *orthologs have overlapping function during morphogenesis (Table 2), indicating that the functional relationship between this pair of genes has been selectively maintained for over 100 million years. This observation suggests that the overlapping functions of other pairs of T-box genes, which are likely to be common throughout the Metazoa, may also be conserved.

### A muscle differentiation module

**Figure 3:**
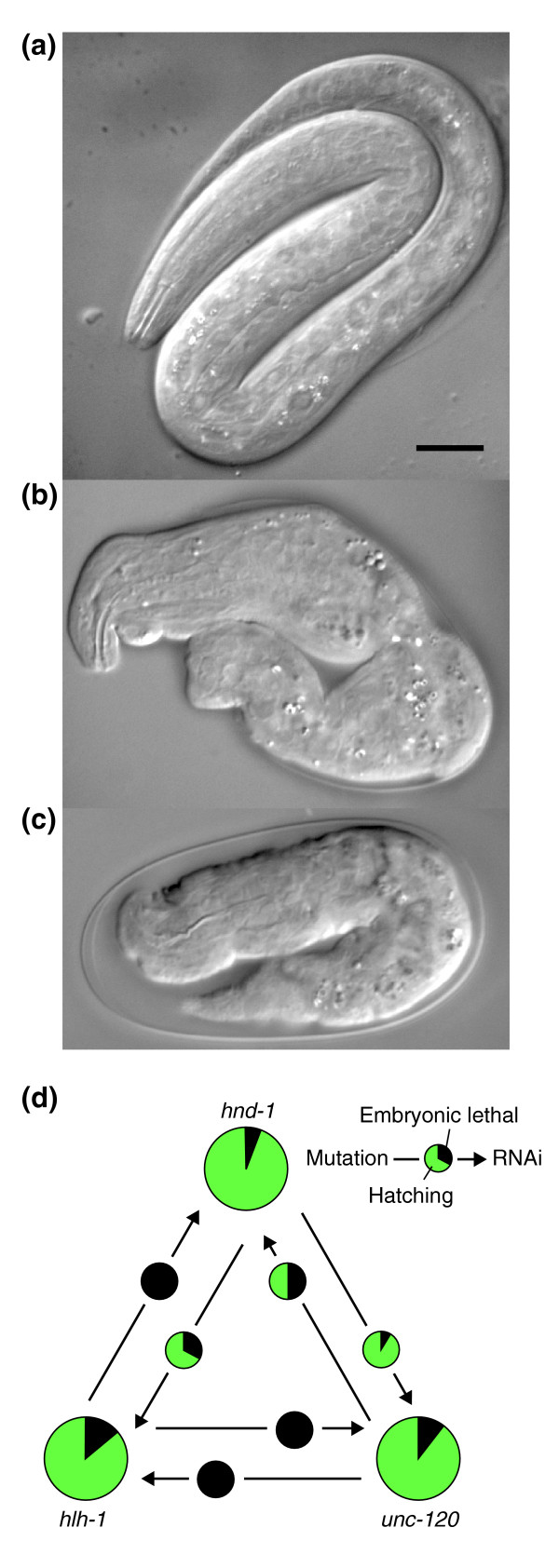
Modular genetic interactions between three transcription factors controlling differentiation of muscle. A recently hatched L1 larva is shown for **(a) **wild type and **(b) ***hlh-1(cc561)*, and **(c) **an arrested Pat embryo is shown for *hlh-1(cc561)*; *unc-120(RNAi)*. The scale bar in (a) equals 10 μm and applies to (a-c). Mutation of *hlh-1*, *hnd-1 *or *unc-120 *alone results in dumpy, uncoordinated larvae and Pat embryos (as in (b) and (c)) at low frequency. Disruption of function of any two of these three genes by mutation and RNAi significantly elevates both frequencies so that in the most potent combinations 100% Pat embryos result. The Pat phenotype has been shown to result specifically from the disruption of muscle differentiation and function [26,27]. **(d) **A summary of the proportion hatching (green) and proportion arresting as Pat embryos (black) for each single mutant and each of the six genetic interactions tested.

It has been suggested that modules of genes comprise units of biological function [23]. Because they operate in concerted fashion, genes in a module are expected to have increased likelihood of being co-expressed and sharing physical and genetic interactions relative to a random set of genes. Clustering a large synthetic lethal matrix in *S. cerevisiae *identified multiple functional modules [9], and we identify a module in our matrix around the *hlh-1 *gene (Figure 1). We detect five genetic interactions (*P *< 0.001) out of six tested among *hlh-1*, *hnd-1*, and *unc-120*. All three genes encode transcription factors expressed exclusively in the muscle progenitor cells of the early embryo [13,24,25]. Disruption of function by mutation or RNAi of any one of the three genes results in a low frequency of embryonic lethality with embryos arresting paralyzed at the two-fold stage (Pat) (Figure 3). Embryos require muscle contraction to elongate and pass from the two-fold to three-fold embryonic stage, and the Pat phenotype results from a complete failure of muscle differentiation [26,27]. A large fraction of animals that do hatch after disruption of *hlh-1*, *unc-120*, or *hnd-1 *show a less severe phenotype (uncoordinated, dumpy larvae) likely reflecting partial muscle function [26,27] (Figure 3). However, disruption of function of any two of the three genes significantly elevates the frequency of Pat embryos, with disruption of *hlh-1 *and *unc-120 *resulting in 100% Pat embryos (Figure 3). The fact that *hlh-1*, *unc-120*, and *hnd-1 *have identical individual and synthetic phenotypes, in addition to their identical early embryonic expression patterns, indicate that they comprise a muscle-differentiation module. Although fewer genes define this module than those identified in larger screens in yeast [9], the specificity of its defining phenotype indicates that *hlh-1*, *unc-120*, and *hnd-1 *function in concert, and it is likely that future synthetic screens will identify additional members of the module.

If both RNAi and genetic disruptions of gene function are null, the synthetic lethal matrix should be symmetric. However, we can only test the pair of reciprocal interactions when mutations of both genes are available and RNAi of neither gene results in 100% lethality in wild type. Although there is some symmetry in the matrix around *hlh-1 *(Figure 1), the interactions detected are not all reciprocal (Figure 3). The efficiency of RNAi varies from gene to gene and often fails to result in null phenotypes, suggesting a significant number of false negatives among the interactions tested and explaining the imperfect symmetry among the interactions detected. The pattern of interactions defining the muscle module suggests that *hlh-1 *is the most essential of the three genes, or that its function is the most potent, and *hnd-1 *the least (Figure 3), a model that accounts for the reported lack of genetic interaction between heterozygous *hlh-1 *and homozygous *hnd-1 *mutations [25].

The functional importance of the muscle module we identified is underscored by its conservation. Well characterized genetic interactions exist among the vertebrate homologs of *hlh-1 *- the myogenic basic helix-loop-helix (bHLH) regulatory factors MyoD, myf5, myogenin, and MRF4 [28]. In addition, interactions between vertebrate myogenic bHLH genes and homologs of *unc-120 *(the MEF2 group of MADS-box regulators) have been described [29]. Interactions among and between vertebrate myogenic bHLH and MADS-box regulators is likely to be the result of cooperative activation of common target genes as well as their ability to activate each other transcriptionally [28-30]. It is possible that *hlh-1*, *hnd-1*, and *unc-120*, like their vertebrate counterparts, display modular genetic interactions because they share common target genes, and they may also activate each other transcriptionally.

## Conclusions

We show that the facility of RNAi in *C. elegans *can be used to screen a large number of genes for synthetic phenotypes. Using an unbiased experimental approach we identified both easy-to-predict genetic interactions between similar co-expressed genes and difficult-to-predict genetic interactions between non-homologous genes. We also used reverse genetics in *C. briggsae *to show that genetic interaction between a pair of co-expressed homologous transcription factors (*tbx-8 *and *tbx-9*) is conserved, suggesting an adaptive role of overlapping function as opposed to the transient result of gene duplication. Furthermore, we identify a genetic module defined by reciprocal interactions among three transcription factors that is required for differentiation of muscle. A similar network among homologous genes is conserved in vertebrates, suggesting that a single master regulator cannot robustly control muscle development. Given the wealth of existing mutants and the ease of RNAi in *C. elegans*, our approach can be scaled up to identify the core set of metazoan developmental genetic interactions.

## Materials and methods

DNA templates for *in vitro *transcription were prepared by amplification of 100-1000 base pair (bp) sequences from cDNA by PCR. PCR primers were designed to amplify unique sequences in order to ensure specificity of RNAi (Additional data file 3), and a minimal T7 promoter sequence was added to the 5' end of both primers. PCR DNA was purified on a DNA Clean and Concentrator-5 column (Zymo Research), and a high-yield *in vitro *transcription kit (Promega) was used to produce dsRNA. The dsRNA was reannealed by heating to 90°C and then cooling 6°C/min to 25°C before organic extraction with a 1:1 mixture of phenol and chloroform. Following ethanol precipitation, dsRNA was dissolved in Soaking Buffer [31] at high concentration, which was then measured by spectrophotometer and diluted to 5 mg/ml.

Eight to twelve larval stage 4 (L4) worms were soaked in 0.75 μl 5 mg/ml dsRNA for 24 h at 20°C and then transferred to a recovery plate with *Escherichia coli *strain OP50 for 12 h at 25°C and then transferred to a score plate with OP50. After 12 h at 25°C on the score plate, adults were removed, and after another 24 h at 25°C, embryos and larvae were counted under a dissecting microscope (with the exception of *unc-120(st364) *which is temperature-sensitive and was therefore scored at 15°C). For strains carrying viable alleles, embryonic lethality was scored for the homozygous progeny of homozygous parents. For strains carrying 100% lethal alleles, embryonic lethality was scored for the mixed progeny of either heterozygous or hemizygous parents. For RNAi in *C. briggsae*, 1 mg/ml dsRNA was injected into the germline of young wild-type adults (strain AF16), and the worms were rescued and scored in the same way as the soaked *C. elegans*.

For statistical analysis of the data, percent embryonic lethality was converted to survival (1 - % embryonic lethality). All survival values were normalized by the survival of N2 (wild type) in Soaking Buffer (no dsRNA). For each genetic interaction tested, the null hypothesis used is that the survival of the double disruption (mutation and RNAi) equals the product of survivals for each single disruption (mutation alone × RNAi alone). The pairs of duplicate survival measurements for each single disruption are multiplied to generate four values for the expected survival of the double disruption, and a *t*-test is used to determine if the observed survival (*n *= 2) is significantly different from expected (*n *= 4). For example, if two genes function independently, and the survival following disruption of each alone is 90%, then our model expects 81% survival following double disruption since the 90% of individuals surviving one disruption have a 10% chance of dying from the other disruption. If survival is significantly different from the expected 81% then we conclude that the two genes interact.

For microscopy, embryos were mounted on 2% agar pads under coverslips and allowed to develop in a humid box for 12-15 h at 25°C. A Zeiss Axiophot with 100× differential interference contrast optics was used and digital images captured.

## Additional data files

The following additional data are available with the online version of this paper. Additional data file 1 contains replicate embryonic lethality measurements and corresponding *P*-values for synthetic lethality. Additional data file 2 contains data showing that double RNAi by soaking worms in dsRNA for two genes at once is an inefficient means of synthetic genetic analysis. Additional data file 3 lists primer sequences used in this study to amplify coding sequences from cDNA for use as templates for dsRNA.

## Supplementary Material

Additional File 1 Replicate embryonic lethality measurements and corresponding *P*-values for synthetic lethality. Replicate embryonic lethality measurements and corresponding *P*-values for synthetic lethality. Click here for file

Additional File 2 Double RNAi by soaking worms in dsRNA for two genes at once is an inefficient means of synthetic genetic analysis. Percent embryonic lethality measured by both double RNAi and RNAi in mutant backgrounds is presented for comparison. Each column corresponds to RNAi of a given gene, and each row corresponds to either RNAi or a mutant allele of a given gene. Each value corresponds to a specific combination of perturbations, either RNAi of two genes or RNAi and mutation, with the controls as exceptions. A genetic interaction is detected for the highly homologous genes *tbx-8 *and *tbx-9 *with both methods, but none of the strong interactions between the three relatively non-homologous myogenic regulators (*hlh-1*, *unc-120*, and *hnd-1*) are detected by double RNAi. Each value presented is the average of two independent experiments; at least 100 progeny were counted for each experiment. dsRNA concentration was 5 mg/ml for all experiments. NA stands for not applicable. Click here for file

Additional File 3 A table listing primer sequences used in this study to amplify coding sequences from cDNA for use as templates for dsRNA. A minimal T7 promoter sequence (TAATACGACTCACTATAGGG) was added to the 5' end of each of the gene-specific sequences listed in the table so that PCR products could be used as *in vitro *transcription templates to make dsRNA. Click here for file

## Figures and Tables

**Table 1:** Genes included in the synthetic lethality matrix

ORF	Gene	Identification	Genotype used in this study
F17A2.5	*ceh-40*	Homeodomain protein (*Extradenticle *ortholog)	*ceh-40(ok740) *X
K10B4.6	*cwn-1*	Putative Wnt ligand	*cwn-1(ok546) *II
D1081.2	*unc-120*	MADS domain TF	*unc-120(st364) *I
B0304.1	*hlh-1*	bHLH TF	*hlh-1(cc561) *II
C44C10.8	*hnd-1*	Hand bHLH TF	*hnd-1(q740) *X
F35G12.6	*mab-21*	Highly conserved novel protein	*mab-21(bx53)*III; *him-5(e1490)*V
F11C1.6	*nhr-25*	Nuclear hormone receptor	+/szT1 [*lon-2(e678)*] I; *nhr-25(jm2389)/*szT1 X
Y75B8A.2	*nob-1*	Homeodomain TF (posterior Hox paralog)	*nob-1(ct223) dpy-18(e364) unc-25(e156) *III; eDp6 (III;f)
K02B9.4	*elt-3*	GATA TF	*elt-3(gk121) *X
T07C4.2	*tbx-8*	T-box TF (Brachyury)	*tbx-8(ok656) *III
M142.4	*vab-7*	Homeobox TF (even-skipped subfamily)	*vab-7(e1562) *III
T07C4.6	*tbx-9*	T-box TF (Brachyury)	Mutant not available
C55C2.1		Zinc-finger TF	Mutant not available
C38D4.6	*pal-1*	Homeobox TF (cad subfamily)	*pal-1(ct224) dpy-17(e164) ncl-1(e1865) unc-36(e251*) III; sDp3(f:III); *lin-2(e1309) *X
W09C2.1	*elt-1*	GATA TF	*elt-1(zu180) unc-43(e408*)/*unc-24(e138) dpy-20(e1282) *IV
F18A1.2	*lin-26*	Zinc-finger TF	*lin-26(n156) *II
T28F12.2	*unc-62*	Meis-class homeodomain (*Homothorax *ortholog)	*unc-62(e644) *V
C28C12.7	*spp-10*	Predicted prosaposin	Mutant not available
R02D3.1		Dehydrogenase	Mutant not available
C09D4.2		Uncharacterized	Mutant not available
R07C3.11		Uncharacterized	Mutant not available
T22B7.3		Uncharacterized	Mutant not available
C46H11.2		Uncharacterized	Mutant not available
ZK1307.1		Uncharacterized	Mutant not available
C01G6.1	*aqp-2*	Aquaporin	Mutant not available
T27D12.1		Sodium/phosphate transporter	Mutant not available

Most of the genes were included in the matrix because they had been genetically determined to be PAL-1 targets (that is, their expression either directly or indirectly depends on PAL-1 function) and were therefore presumed to have genetic interactions among them. Three additional genes encoding transcriptional regulators (*lin-26*, *ceh-40*, and *unc-62*) were included as their phenotypes and expression patterns suggest that they may have genetic interactions with PAL-1 targets. TF, transcription factor.

**Table 2:** Functional redundancy of *tbx-8 *and *tbx-9 *is conserved in *C. briggsae*

Genotype	*C. elegans*			*C. briggsae*		
	Embryonic lethality	Morphological defect	Wild type larvae	Embryonic lethality	Morphological defect	Wild type larvae

*tbx-8(RNAi)*	1%	1%	98%	4%	1%	96%
*tbx-9(RNAi)*	1%	1%	98%	3%	2%	95%
*tbx-8(RNAi)*;*tbx-9(RNAi)*	48%	52%	0%	39%	46%	15%

RNAi was used for both genes in both species as no mutations are available for *C. briggsae*.
