# Supplementary material for: Synthetic lethal analysis of Caenorhabditis elegans posterior embryonic patterning genes identifies conserved genetic interactions
Source: Genome Biol. 2005 Apr 11;6(5):R45. doi: 10.1186/gb-2005-6-5-r45 (PMC1175957; doi:10.1186/gb-2005-6-5-r45)
Supplement: Additional File 1 — Replicate embryonic lethality measurements and corresponding P-values for synthetic lethality. Replicate embryonic lethality measurements and corresponding P-values for synthetic lethality. [file gb-2005-6-5-r45-S1.doc]

Supplementary Table 1.

|  | Wild-Type | *ceh-40* | *cwn-1* | *unc-120* | *hlh-1* | *hnd-1* | *mab-21* | *nhr-25* | *nob-1* | *elt-3* | *tbx-8* | *vab-7* | *pal-1* | *elt-1* | *lin-26* | *unc-62* |
| --- | --- | --- | --- | --- | --- | --- | --- | --- | --- | --- | --- | --- | --- | --- | --- | --- |
| Soaking Buffer | 0%, 1% | 1%, 0% | 2%, 1% | 8%, 12% | 13%, 13% | 8%, 3% | 23%, 30% | 68%, 77% | 64%, 57% | 1%, 0% | 20%, 27% | 5%, 5% | 67%, 64% | 27%, 31% | 3%, 11% | 18%, 21% |
| *ceh-40* | 3%, 0% | 1%, 0% 0.4165 | 0%, 4% 0.799254 | 3%, 1% 0.011741 | 12%, 29% 0.289788 | 6%, 0% 0.302421 | 14%, 18% 0.030926 | 69%, 68% 0.323425 | 54%, 61% 0.44089 | 0%, 1% 0.536924 | 19%, 22% 0.372697 | 4%, 5% 0.48443 | 70%, 55% 0.503518 | 26%, 34% 0.950633 | 0%, 0% 0.090034 | 30%, 38% 0.009833 |
| *cwn-1* | 2%, 0% | 1%, 0% 0.788464 | 1%, 1% 0.350667 | 12%, 10% 0.948735 | 20%, 21% 0.00144 | 5%, 4% 0.625028 | 23%, 23% 0.26218 | 75%, 65% 0.640444 | 65%, 65% 0.270169 | 1%, 1% 0.793033 | 36%, 33% 0.032476 | 2%, 4% 0.121621 | 69%, 61% 0.74218 | 32%, 27% 0.909374 | 6%, 3% 0.461493 | 31%, 36% 0.003637 |
| *unc-120* | 4%, 2% | 2%, 0% 0.16053 | 0%, 7% 0.810578 | 9%, 26% 0.426228 | 99%, 100% 0 | 8%, 9% 0.726391 | 17%, 27% 0.192904 | 76%, 78% 0.414555 | 56%, 80% 0.495107 | 1%, 0% 0.049856 | 40%, 36% 0.019711 | 9%, 3% 0.518649 | 59%, 73% 0.857824 | 31%, 35% 0.444002 | 9%, 4% 0.439487 | 43%, 31% 0.016959 |
| *hlh-1* | 3%, 5% | 8%, 5% 0.170824 | 10%, 18% 0.021383 | 99%, 99% 0.000001 | 19%, 45% 0.114901 | 34%, 31% 0.000503 | 15%, 21% 0.034926 | 82%, 73% 0.470691 | 84%, 72% 0.034347 | 15%, 22% 0.003494 | 43%, 51% 0.006262 | 5%, 4% 0.019516 | 77%, 71% 0.041479 | 42%, 54% 0.014569 | 35%, 40% 0.001608 | 40%, 51% 0.003087 |
| *hnd-1* | 2%, 0% | 6%, 5% 0.026077 | 2%, 3% 0.830418 | 73%, 59% 0.000312 | 99%, 100% 0 | 5%, 8% 0.723433 | 15%, 22% 0.091927 | 72%, 69% 0.615723 | 62%, 60% 0.961876 | 3%, 4% 0.044683 | 37%, 25% 0.210991 | 16%, 13% 0.00246 | 60%, 72% 0.945041 | 36%, 33% 0.094755 | 0%, 5% 0.267027 | 46%, 46% 0.000079 |
| *mab-21* | 2%, 1% | 1%, 1% 0.443245 | 2%, 2% 0.666101 | 1%, 5% 0.019405 | 17%, 19% 0.010529 | 3%, 4% 0.259585 | 24%, 15% 0.139431 | 66%, 73% 0.482365 | 51%, 60% 0.278611 | 1%, 1% 0.553431 | 17%, 18% 0.115382 | 3%, 5% 0.146606 | 66%, 45% 0.192402 | 32%, 39% 0.097612 | 0%, 5% 0.241569 | 26%, 42% 0.058048 |
| *nhr-25* | 1%, 1% | 3%, 2% 0.06961 | 3%, 1% 0.994494 | 9%, 14% 0.655976 | 17%, 19% 0.001296 | 6%, 7% 0.555751 | 23%, 28% 0.696885 | 73%, 68% 0.629553 | 70%, 54% 0.827904 | 2%, 1% 0.038707 | 38%, 39% 0.008842 | 4%, 6% 0.755826 | 62%, 57% 0.035054 | 22%, 30% 0.337407 | 53%, 31% 0.009714 | 35%, 41% 0.001306 |
| *nob-1* | 37%, 29% | 54%, 59% 0.003069 | 30%, 25% 0.154958 | 33%, 34% 0.12668 | 33%, 26% 0.025606 | 27%, 21% 0.031562 | 55%, 42% 0.707788 | 80%, 86% 0.644364 | 75%, 80% 0.227397 | 40%, 54% 0.061483 | 52%, 56% 0.206114 | 32%, 30% 0.207251 | 86%, 74% 0.521478 | 60%, 68% 0.031053 | 58%, 58% 0.006007 | 45%, 33% 0.208081 |
| *elt-3* | 2%, 0% | 1%, 0% 0.427588 | 0%, 2% 0.520017 | 6%, 4% 0.031 | 19%, 13% 0.344942 | 9%, 8% 0.249627 | 24%, 18% 0.174347 | 75%, 68% 0.832743 | 61%, 63% 0.824049 | 0%, 4% 0.436185 | 35%, 23% 0.353344 | 5%, 5% 0.889195 | 59%, 56% 0.007049 | 27%, 33% 0.896925 | 4%, 11% 0.943094 | 17%, 41% 0.300417 |
| *tbx-8* | 5%, 1% | 1%, 0% 0.283423 | 2%, 4% 0.612959 | 5%, 10% 0.143119 | 26%, 12% 0.511258 | 36%, 16% 0.056167 | 19%, 21% 0.066833 | 70%, 70% 0.484984 | 58%, 60% 0.510932 | 1%, 3% 0.671585 | 40%, 33% 0.046714 | 8%, 7% 0.958747 | 70%, 63% 0.953895 | 30%, 28% 0.513822 | 4%, 10% 0.544161 | 36%, 33% 0.005407 |
| *vab-7* | 0%, 0% | 0%, 1% 0.652257 | 1%, 3% 0.204969 | 10%, 8% 0.681794 | 8%, 12% 0.079409 | 20%, 8% 0.085264 | 27%, 22% 0.596844 | 70%, 69% 0.482233 | 61%, 54% 0.488216 | 1%, 0% 0.052343 | 32%, 37% 0.034125 | 2%, 7% 0.965863 | 70%, 68% 0.06399 | 34%, 34% 0.039567 | 4%, 18% 0.433951 | 23%, 24% 0.028862 |
| *tbx-9* | 1%, 1% | 0%, 1% 0.401459 | 1%, 0% 0.116114 | 6%, 5% 0.043873 | 9%, 6% 0.001951 | 3%, 2% 0.14541 | 14%, 15% 0.017502 | 74%, 85% 0.256244 | 55%, 51% 0.101615 | 1%, 1% 0.041433 | 73%, 77% 0.000103 | 10%, 3% 0.583277 | 74%, 65% 0.32628 | 35%, 29% 0.40772 | 2%, 8% 0.614592 | 30%, 31% 0.00164 |
| C55C2.1 | 0%, 3% | 0%, 0% 0.326308 | 1%, 1% 0.24827 | 2%, 4% 0.016381 | 14%, 12% 0.308435 | 14%, 9% 0.109562 | 22%, 13% 0.076363 | 67%, 68% 0.256262 | 42%, 53% 0.05092 | 1%, 0% 0.594368 | 20%, 23% 0.491091 | 7%, 4% 0.883312 | 72%, 60% 0.913456 | 33%, 22% 0.56573 | 12%, 17% 0.145446 | 18%, 31% 0.413868 |

Supplementary Table 1. Replicate embryonic lethality measurements and corresponding P-values for synthetic lethality.
