# Supplementary material for: Synthetic lethal analysis of Caenorhabditis elegans posterior embryonic patterning genes identifies conserved genetic interactions
Source: Genome Biol. 2005 Apr 11;6(5):R45. doi: 10.1186/gb-2005-6-5-r45 (PMC1175957; doi:10.1186/gb-2005-6-5-r45)
Supplement: Additional File 2 — Double RNAi by soaking worms in dsRNA for two genes at once is an inefficient means of synthetic genetic analysis. Percent embryonic lethality measured by both double RNAi and RNAi in mutant backgrounds is presented for comparison. Each column corresponds to RNAi of a given gene, and each row corresponds to either RNAi or a mutant allele of a given gene. Each value corresponds to a specific combination of perturbations, either RNAi of two genes or RNAi and mutation, with the controls as exceptions. A genetic interaction is detected for the highly homologous genes tbx-8 and tbx-9 with both methods, but none of the strong interactions between the three relatively non-homologous myogenic regulators (hlh-1, unc-120, and hnd-1) are detected by double RNAi. Each value presented is the average of two independent experiments; at least 100 progeny were counted for each experiment. dsRNA concentration was 5 mg/ml for all experiments. NA stands for not applicable. [file gb-2005-6-5-r45-S2.doc]

Supplementary Table 2.

|  | Soaking Buffer | *tbx-8(RNAi)* | *tbx-9(RNAi)* | *hlh-1(RNAi)* | *unc-120(RNAi)* | *hnd-1(RNAi)* |
| --- | --- | --- | --- | --- | --- | --- |
| Wild-type | 1% | 1% | 1% | 3% | 2% | 2% |
| *tbx-8(RNAi)* | 1% | NA | 45% | 2% | 2% | 2% |
| *tbx-8(ok656)* | 27% | 40% | 83% | 43% | 36% | 37% |
| *hlh-1(RNAi)* | 3% | 2% | 5% | NA | 2% | 4% |
| *hlh-1(cc561)* | 13% | 11% | 9% | 45% | 100% | 99% |
| *unc-120(RNAi)* | 2% | 2% | 3% | 4% | NA | 1% |
| *unc-120(st364)* | 9% | 10% | 10% | 99% | 11% | 54% |
| *hnd-1(RNAi)* | 2% | 2% | 2% | 4% | 1% | NA |
| *hnd-1(q740)* | 3% | 7% | 7% | 15% | 5% | 2% |

Supplementary Table 2. Double RNAi by soaking worms in dsRNA for two genes at once is an inefficient means of synthetic genetic analysis. Percent embryonic lethality measured by both double RNAi and RNAi in mutant backgrounds is presented for comparison. Each column corresponds to RNAi of a given gene, and each row corresponds to either RNAi or a mutant allele of a given gene. Each value corresponds to a specific combination of perturbations, either RNAi of two genes or RNAi and mutation, with the controls as exceptions. A genetic interaction is detected for the highly homologous genes *tbx-8* and *tbx-9* with both methods, but none of the strong interactions between the three relatively non-homologous myogenic regulators (*hlh-1*, *unc-120*, and *hnd-1*) are detected by double RNAi. Each value presented is the average of two independent experiments; at least 100 progeny were counted for each experiment. dsRNA concentration was 5 mg/ml for all experiments. NA stands for not applicable.
