# Supplementary material for: Synthetic lethal analysis of Caenorhabditis elegans posterior embryonic patterning genes identifies conserved genetic interactions
Source: Genome Biol. 2005 Apr 11;6(5):R45. doi: 10.1186/gb-2005-6-5-r45 (PMC1175957; doi:10.1186/gb-2005-6-5-r45)
Supplement: Additional File 3 — A table listing primer sequences used in this study to amplify coding sequences from cDNA for use as templates for dsRNA. A minimal T7 promoter sequence (TAATACGACTCACTATAGGG) was added to the 5' end of each of the gene-specific sequences listed in the table so that PCR products could be used as in vitro transcription templates to make dsRNA. [file gb-2005-6-5-r45-S3.doc]

Supplementary Table 3.

| Gene | Primer 1 | Primer 2 |
| --- | --- | --- |
| *aqp-2* | TTCGTAAAGAGTTGCTCCGC | CGGCTTCTTCATCGACAGTT |
| C09D4.2 | CCGTTAATTTCCACCTCAGC | TGCTTCGAAGTGGATACTTTCA |
| C46H11.2 | GTCGGAGGAACATGGGTCTA | TTCGAAAGCGGACTCTTCAT |
| C55C2.1 | CGTACGAAGTTCCACCGTTC | ACTTCGATTCCTCGTGCTTC |
| *Cb-tbx-8* | | GGCAGAAAAATGTTCCCAAA | | --- | | | CCGTCACGGCTATAAACTCC | | --- | |
| *Cb-tbx-9* | ATGGGCAGTGTCTGGAAAAG | AAAGCTCCAGAAGCCAACAA |
| *ceh-40* | TTTATGAACAAGATGTCCGGT | CCTGTTTGACCTCTTCCGAC |
| *cwn-1* | CCGGGAACGTCATGTAAATC | TCGTTTCTGACATGGCTCAC |
| *elt-1* | CTACGAAGGAAAACCCGTTG | TTCTTCATTCTTCGCGATCC |
| *elt-3* | TGCGGACTTCACTAATGCAC | CGTGTCCATGTCTCTGATGG |
| *hlh-1* | GTGTCACCGCAAATGACATC | GGAGTTGTTCGTCGGTCATT |
| *hnd-1* | CCCACAATCAGGAGTTGGAT | GCGAGAACTGATTTGGGAAG |
| *lin-26* | TCATCAAGTCCCGAATCTCC | TGTTGGCTTTCCACAGAGTG |
| *mab-21* | TCGAGTAGATGACAAGTACACCG | ACATGTTCTCTGCACCATGC |
| *nhr-25* | TGAAAGTTGCAAGGGCTTCT | GGGAGAATTGGTGATGTGCT |
| *nob-1* | CCGTTCGGAAAGAGAATCTG | CTTTCCGCTGAATACTGCCT |
| *pal-1* | GGTCGATGTCAAGTCGGATT | TCTGTTTGTCACGACGATCC |
| R02D3.1 | GCGGTATGATGCGACAAATA | ATTACTGATGCGTATGGGGC |
| R07C3.11 | CATGCATATTGAGCGATTGG | TATCCGCATTGGACTCCTTC |
| *spp-10* | CGGAATGATGTGCAGAGAGA | GGAAATATCCGAGGATACCCA |
| T22B7.3 | GAGTGAACCGAGAAAAAGCG | TTGAAGAGTGCAGCATTTGG |
| T27D12.1 | ACGCGATTCGTGATTATGGT | GTTGAACGTAGGCGACCAGT |
| *tbx-8* | ATCAGGATAAGCTGTGGAACC | AAAATTGGCATGGGCAATAG |
| *tbx-9* | ACGCGTACAACGAGGAGTTC | AGCGAATGGAAATTGGACTG |
| *unc-120* | ACTCCAACACCATCCTCGTC | ACTCCAACACCATCCTCGTC |
| *unc-62* | TCGACGACGAATTGTACAGC | ATTCGTGTGGTGCTGAGAGA |
| *vab-7* | AGGTCCCATCACTTGTGGAG | AAGGCGAGGGAGAACTTGCG |
| ZK1307.1 | TGTCTGCTGCAAAATCTGCT | TGCTTGAGAGACACCGATTG |

Supplementary Table 3. Gene-specific primer sequences used in this study to amplify coding sequences from cDNA. A minimal T7 promoter sequence (TAATACGACTCACTATAGGG) was added to the 5’ end of each of the gene-specific sequences listed in the table so that PCR products could be used as in vitro transcription templates to make dsRNA.
